# Along with the privilege of authorship come important responsibilities

**DOI:** 10.1186/s12916-014-0214-2

**Published:** 2014-10-24

**Authors:** David Moher

**Affiliations:** Knowledge Synthesis Group, Clinical Epidemiology Program, Ottawa Hospital Research Institute, The Ottawa Hospital, General Campus, 501 Smyth Rd, Room L1288, Ottawa, ON K1H 8L6 Canada; Department of Epidemiology and Community Medicine, University of Ottawa, Roger Guindon Hall, Room 3105, 451 Smyth Road, Ottawa, Ontario K1H 8M5 Canada

**Keywords:** Authorship, Transparency, Responsibility, Quality

## Abstract

Transparently documenting who and why a person is an author of a clinical trial report, as with any other article, is important since it allows research team members appropriate recognition and also likely helps reduce or avoid problems such as ghost authorship. Marušić and colleagues have previously proposed a five-step framework for attributing authorship of pharmaceutical company-sponsored clinical trials. The process is short, easily implemented, and can be used in addition to the authorship guidance provided by the International Committee of Medical Journal Editors. For the framework to gain optimal traction it is important that a strong implementation plan is developed and carried out across a broad spectrum of stakeholders. Authorship brings with it important responsibilities; authors must ensure that articles baring their names must be fit for purpose. This will help guarantee an increased value for published reports of clinical trials.

Please see related article: http://www.biomedcentral.com/1741-7015/12/197

## Commentary

“*Complexity is the enemy of transparency*” [1]. Today, *BMC Medicine* publishes another paper on journalology (publication science) [2]. Attributing authorship, and authorship order, is complex and often a ‘black box’ for prospective authors. Professor Marušić and colleagues have tried to peel back the black box concerning the assigning of authorship for industry-sponsored clinical trials. Their methods are good and reported in sufficient detail to allow interested readers to replicate them [3]. The research team have used an integrated knowledge translation approach to developing their proposed five-steps for transparently disclosing authorship. Participants from pharmaceutical companies that conduct clinical trials, academics, editors, and the Medical Publishing Insights and Practices initiative were involved in the entire process; this facilitates buy-in and support for the process and outcome. These same people are likely to become front-line ambassadors and early adopters for disseminating and implementing the five-step framework within their own working environments, and hopefully, more broadly.

What is positive about this research is that the proposed attribution process for authorship is brief, and not complex; it’s only five-steps. It is meant to augment the guidance provided by the International Committee of Medical Journal Editors [4]. To enhance uptake of the framework it will be important for the team, or others, to develop a bank of worked examples for each step in the five-step process. Using worked examples from specific trials will likely facilitate implementation. The authors have started the process with seven case examples included in their publication. A dedicated website for the framework whereby authorship examples can be submitted by pharmaceutical companies and others, vetted and added to a bank of examples, freely accessible to anybody, is worth considering. This will help prospective clinical trialists ensure a transparent process in deciding on authorship.

If this authorship initiative is to be successful it requires endorsement and, more importantly, implementation. As Marušić and colleagues note [2], previous efforts, such as contributorship, have not been broadly implemented. What is less clear is how the proposed framework is going to be endorsed and implemented. Important initial steps should include strong and consistent language of endorsement across all of the pharmaceutical companies involved in the development of the five-step process. Support and endorsement from umbrella groups, such as the Pharmaceutical Research and Manufacturers of America [5], and others such as CONSORT [6], is also worth considering. While endorsement is a useful step, it is difficult to measure and likely not the most relevant outcome. More important will be to develop plans based on appropriately developed approaches [7,8] to implement the framework. This is likely to be most effective when pharmaceutical companies modify their authorship practices and polices when conducting any clinical trial. An effective policy would require all clinical trials to implement the five-step framework at their inception. Without strong implementation the framework is less likely to affect positive change. This has been observed when trying to implement reporting guidelines in biomedical journals [9,10]. Part of any implementation plan also needs to include an evaluation of the framework. It is important to collect data that will inform its usefulness. There is little merit in maintaining policies that are not supported by evidence.

Marušić et al. are silent on whether their framework can be used when developing authorship for submitting clinical trial protocols for publication consideration [2]. Making clinical trial protocols accessible is important and at least one Biomed Central journal – *Trials* – regularly publishes them. Additionally, with the requirement of trial registration, this framework could also be used when completing the investigator information part of the registration.

Most of us are not born authors. It is an acquired skill that often starts during graduate school. This is where all journalology issues, including those pertaining to authorship issues (e.g., attributing authorship, authorship order, and ghost and guest authorship, author responsibilities) should be formally taught and discussed. Developing such skills early can translate into something useful throughout a researcher’s (author’s) career. It is unfortunate that almost all universities, and other centres of higher learning, appear to have abdicated their responsibilities regarding formally teaching journalology; the irony is not lost on me. These institutions are the very same places developing the next generation of biomedical researchers. Universities need to set aside appropriate resources to enable and promote such courses, and others, related to journalology [11].

While authors have rights and privileges, they also have important responsibilities that require much greater attention. Given the opportunity of authorship, it is equally important to assert this responsibility. Authors must ensure that papers baring their name are “fit for purpose” [12]. Here, authors need to ensure that every report baring their name is a completely reported and transparent account of what was done (methods) and found (results) to enable interested readers to replicate the methods and use the results. Collectively, authors have not performed appropriately with regards to reporting their clinical trials. This avoidable waste is troublesome for shareholders of publicly traded pharmaceutical companies and tax payers of publicly-funded clinical trials. It is not a good return of a fiscal investment when reports of trials are so inadequate that their results cannot be used. For example, Duff et al. [13] examined 262 reports of trials from the most prominent oncology journals assessing them for 10 essential elements regarding the description of their interventions, such as the drug’s name and route of administration. The authors reported that only 11% of the articles reported all 10 characteristics. Although we have seen improvements over time in reporting the unique characteristics of randomized trials – sequence generation, allocation concealment, and implementation – these items are adequately reported in less than half of the trial reports [14]. In some clinical specialties, the situation is much worse [15].

Disclosing authorship transparently is important for any manuscript being submitted to a biomedical journal for publication consideration. The responsibilities associated with authorship must be taken seriously. This might help increase value and reduce avoidable waste of biomedical research.
